# Working the Edge: The Emotional Experiences of Commissioning and Funding Arrangements for Service Leaders in the Sexual Violence Voluntary Sector

**DOI:** 10.1177/10778012241239945

**Published:** 2024-03-21

**Authors:** Clare Gunby, Louise Isham, Harriet Smailes, Caroline Bradbury-Jones, Sarah Damery, Jenny Harlock, Fay Maxted, Deb Smith, Julie Taylor

**Affiliations:** 1 School of Nursing and Public Health, 5289 Manchester Metropolitan University, Manchester, UK; 2 Institute of Applied Health Research, 1724 University of Birmingham, Birmingham, UK; 3 Department of Social Work and Social Care, 1724 University of Birmingham, Birmingham, UK; 4 School of Criminology, 4488 University of Leicester, Leicester, UK; 5 School of Nursing, 1724 University of Birmingham, Birmingham, UK; 6 Warwick Medical School, 2707 University of Warwick, Warwick, UK; 7 The Survivors Trust, Rugby, UK; 8 1729 Birmingham Women's and Children's Hospital NHS Foundation Trust, Birmingham, UK

**Keywords:** commissioning, edgework, emotion, sexual violence, specialist voluntary sector

## Abstract

The specialist voluntary sector plays a crucial role in supporting survivors of sexual violence. However, in England, short-term funding underpins the sector's financial stability. This article examines sector leaders’ ways of coping, resisting and being affected by funding practices. Using the concept of edgework, we show how funding and commissioning dynamics push individuals to the edge of service sustainability, job satisfaction, and emotional well-being. We examine how these edges are “worked,” for example, by circumventing and remolding the edge. We offer an original way to theorize participants, make visible the emotional toll of service precarity and offer suggestions for support.

## Introduction

The role of the specialist voluntary sector in supporting survivors of sexual violence is often neglected in academic research, with the focus tending to be on state provision. While U.K.-based and international research has examined the emotional toll on voluntary (and statutory) practitioners who work in the field of sexual violence and abuse (Horvath et al., 2021; Horvath & Massey, 2018; Massey et al., 2019; Thornton & Novak, 2010), this has typically focused on the impact of supporting those who have experienced trauma. To our knowledge, no literature has explored the effects associated with managing the sustainability of a voluntary organization. Thus, in this article, we examine the emotional impacts of navigating the unstable, short-term and top-down funding and commissioning context that underpins the status quo for specialist voluntary services in England (All-Party Parliamentary Group: APPG, 2018; Women's Resource Centre: WRC, 2018, 2020).

Drawing on 13 interviews with senior leaders of specialist voluntary sector sexual violence (SVSSV) services, conducted between 2019 and 2020, we examine how operating in a precarious funding and commissioning context is experienced by those leaders. Using the concept of edgework (Lyng, 1990), we apply an original lens to argue that funding and commissioning processes are central to generating an unnerving feeling of “working at the edge.” Or, more specifically, of working at one (or more) specific edges. These include the edge of surviving as a service versus no longer being financially sustainable; the edge of remaining in the sector versus leaving; and the edge of holding on psychologically versus “going under” emotionally. We make visible the ways participants are affected by funding practices, as well as examine how the edge is “worked,” resisted and/or negotiated, to not fall over—but instead—stay in control.

Our focus is on SVSSV services in England and generates insights into how women leaders struggle with, and against, state approaches to the resourcing of sexual violence services. However, due to precarious funding and commissioning arrangements demarcating much of the charitable sector (Harlock, 2014), our findings have implications for other areas of the voluntary sphere and in countries with third-sector organizations. We do, however, argue that for SVSSV services, working with trauma and stigma compounds the emotional impact of funding and commissioning processes.

### Sexual Violence Services, Funding and Commissioning Structures

SVSSV services are defined as those voluntary organizations which have been developed and their primary purpose is to support, someone who is or has been affected by sexual violence (Home Office, 2022). The first Rape Crisis Center in England opened in 1973, emerging out of the consciousness-raising activities of second-wave feminism and informed by the U.S. model (the first Rape Crisis Center having opened in Washington DC the year prior). Services were underpinned by a feminist approach that situated rape as a gendered and structural issue, lobbying for political transformation while also supporting survivors (Jones & Cook, 2008; Vera-Gray, 2020). The late 1970s and 1980s saw the expansion of voluntary provision in England, including the development of services for adult survivors of childhood sexual abuse. SVSSV services can therefore encompass smaller grassroots organizations that emerged in response to local unmet needs, sometimes for specific groups (e.g., children and men), as well as larger services that work with a range of survivors, forms of sexual victimization and that offer a less gendered approach. Black activist critique at the time, however, emphasized that the violence and service response Black and minoritized women faced was mediated by intersecting forms of oppression, explaining why the provision was often unable (or unwilling) to meet their needs (Chantler & Thiara, 2017). Specialist services delivered by and for minoritized women filled this gap and while supporting those who have been subjected to different forms of male violence (not exclusively sexual), between 67% and 76% of service users are seeking help for sexual violence and abuse (Thiara & Roy, 2020).

While the work that happens within SVSSV services varies—funding withstanding—they offer a nonmedicalized, holistic response via medium to longer-term counseling and emotional and/or practical support. Provision is premised on empowering survivors and helping them to challenge thoughts of self-blame (Jones & Cook, 2008). The “difference” services make has been underresearched. However, literature has characterized them as flexible, enabling, holding and mending (Hester & Lilley, 2018), as well as life-changing and life-saving (WRC, 2018).

Funding for SVSSV services in England takes a variety of forms, including donations, grants, and commissioned contracts (APPG, 2018; WRC, 2018). Commissioning refers to the process of funding, planning, and purchasing services on behalf of local communities, with responsibility for the commissioning of sexual violence services having historically lied nationally with the Ministry of Justice, Home Office and/or National Health Service (NHS) England. However, moves toward localism have meant that responsibility for budget-holding and service commissioning has increasingly been devolved to local municipal councils, police and crime authorities (typically, the Office for the Police and Crime Commissioner) and local health planning bodies (that is, Clinical Commissioning Groups (CCGs), which were replaced by Integrated Care Boards in 2022 in England). While grants (which have fewer stipulations on how funds are to be spent) continue to be awarded to SVSSV services by these national and local bodies, increasingly, commissioning takes place through a process of competitive tendering where organizations bid to provide services on behalf of those statutory bodies. This reflects a wider trend toward competitive tendering and procurement processes—and away from grant funding—as the main source of income for the voluntary sector in England. However, public donations and contributions from charitable organizations, trusts, and foundations contribute a significant share (APPG, 2018; WRC, 2018), although regional variation means that funding and commissioning processes vary across England. All of which impacts the existence, size, scope, reach, and quality of local specialist provision (Harlock, 2014).

Indeed, due to funding cuts and commissioning practices, SVSSV services almost halved in England and Wales between the 1980s and 2000s (Westmarland & Alderson, 2013), while a 2009 mapping exercise of the 408 local authorities in England, Scotland, and Wales identified that just one in 10 has a minoritized women's service (Coy et al., 2011). Despite unprecedented financial investment in SVSSV services in response to the pandemic, in 2021, over 10,000 children and adults remained on waiting lists across 39 rape crisis member centers (Rape Crisis England & Wales, RCEW, 2021). This figure does not include approximately 120 other SVSSV services that sit under the membership of The Survivors Trust or those specialist services that work with Black and minoritized women.

Difficulties with service sustainability link to a range of factors including the privileging of short-term grants and contracts for specific services or interventions and the prioritizing of project-based “innovations” over funding ongoing service running costs. Austerity politics has resulted in substantial cuts to commissioner budgets with central government funding for local government bodies falling by 50% between 2010/2011 and 2015/2016 (WRC, 2018). Equally, the shift toward commissioning and away from needs-led grants has affected internal operations and the delivery of services as organizations shift resources toward the demands of competitive tendering and with it, the conferring of additional risks. Competition has increased, “cheapness” favored under the guise of value for money and economies of scale prioritized over social, health, and cultural outcomes and/or specialism. Contracts have been underpriced, service independence and mission have been compromised and smaller SVSSV providers who lack the infrastructure to compete have lost contacts to larger, “mainstream” (i.e., nonspecialist) organizations (APPG, 2018; Home Office, 2022; Ishkanian, 2014; Vacchelli et al., 2015; WRC, 2018, 2020). Commissioners and other service providers do not always see the value of SVSSV services and/or characterize them as “niche” (Westmarland & Alderson, 2013). In turn, there has been a “diminishing of the legitimization” of voluntary organizations as specialists (Vacchelli et al., 2015, p. 109).

### Edgework

Considering this context, it is perhaps surprising that when focusing on the work of SVSSV service practitioners, emphasis has hinged on the burnout, fatigue and/or rewards associated with providing support to survivors (Gunby et al., 2020; Thornton & Novak, 2010), as opposed to the emotional consequences of managing funding and commissioning arrangements. While the challenges of the latter have been described (Donovan & Butterby, 2020; The Survivors Trust, 2020; WRC, 2020), emphasizing their relationship to staff stress and burnout, they have not centered the issue or detailed the means of managing (or otherwise) this financial context. While acknowledging that it would not be possible to separate entirely the emotional impacts of direct work with clients from those which arise from funding and commissioning dynamics, we noted from our data that when stress, sleepless nights, and thoughts of leaving the sector were discussed, these were frequently linked to funding and commissioning practices. It was also evident that these dynamics were pushing participants to the edge: to the psychological edge, the edge of job satisfaction, and the edge of service survival.

Lyng (1990, p. 857) first defined edgework as activities that involve voluntary risk-taking, where there is a “clearly observable threat to one's physical or mental well-being or one's sense of an ordered existence.” The quintessential edgework experience is life-threatening, most easily demonstrated through engagement in extreme activities such as skydiving. The concept, however, has applications beyond physical danger and the edge can be any boundary where an individual may lose control and harm themselves. Specific skills are necessary to survive, to “maintain control over a situation that verges on complete chaos” (Lyng, 1990, p. 859) and which can push individuals to their mental and physical limits. Surviving the edge has been understood as culminating in a physiological high, contextualizing the compulsion to return to the brink (Lyng, 1990). However, edgework is a gendered concept, initially applied to understand male risk-taking behavior and assuming the subjective sensations of the male mind and body (Ward et al., 2020). As the definition has expanded it has begun to capture women's management of the edge, emphasizing that risks are not always practiced voluntarily, but necessitated when doing one's job (Lois, 2001; Lyng, 2009; Ward et al., 2020). As such, there has been a challenge to the linking of edgework to pleasure and increasing recognition of its relationship to emotional pain.

While SVSSV sector leaders have not been situated as edge workers previously, here we make the case that they meet the definition. However, we recognize that in their feminist work to advance the rights of survivors, activist practitioners have long engaged in risk-taking by finding creative means to maneuvre across contexts that minimize and deny the prevalence and significance of sexual violence. In so doing, they have faced threats to their physical and mental well-being and sense of an ordered existence (Lyng, 1990). In other words, SVSSV leaders and practitioners have long been doing edgework, even though their activities have not been characterized as such.

## Methodology

The data presented draws on the first phase of a 3-year NIHR-funded study examining the role, funding, and commissioning of SVSSV services in England (Combes et al., 2019). This initial exploratory, qualitative phase aimed to understand the principal issues shaping the delivery, funding, and commissioning of services. We interviewed service leaders, practitioners, commissioners, and victim-survivors who ran, funded, and used specialist voluntary provision, focusing in this article on the service leader data specifically. Between December 2019 and April 2020, two of the authors (CG and LI) conducted 13 in-depth interviews with senior staff working within an SVSSV service. Informed by a scoping review of academic and gray literature, the interview guide asked about the strengths and limitations of SVSSV services, the relationships, pathways, and comparative differences between voluntary and statutory services and their funding and commissioning arrangements. The guide was tested and refined in two pilot interviews, while a semistructured approach was used to strike the balance between flexibility and comparability (Baillot et al., 2013).

We sought diversity in the size, service-user population, geographic location and funding and commissioning approach of the services we recruited participants. We used our networks, which included Rape Crisis England and Wales and The Survivors Trust—national umbrella organizations for SVSSV services in the United Kingdom—to identify and connect us to individuals from services matching our criteria. CG made email contact and circulated the participant information sheet. Of those individuals approached, 13 women, 12 White, and one Black, working across 11 SVSSV services agreed to participate (while three declined). No individual working within a specialist Black and minoritized women's organization participated and considering the funding context previously set out, this is a limitation of the data.

The services that were represented within the sample were situated within rural, coastal, and urban locations, covering nine counties across the breadth of England. These counties included areas with some of the most ethnically diverse populations in England, while more rural and coastal contexts were significantly less diverse and typically more affluent. The sample was kept small due to the exploratory nature of the research and the recognition that we would go back to recruit from this same population in later phases of data collection. However, the consistency in categories generated across participants working in different locations suggests that we can draw rigorous conclusions about the impacts of funding and commissioning practices on service leaders.

Nine telephone and four face-to-face interviews took place, based on participant preference. Face-to-face interviews occurred at the participant's place of work. Interviews lasted between 54 min and 1 hr 50 min (with an average of 69 min). Telephone interviews were of equivalent length to those conducted in person, suggesting that the mode of delivery did not impact the depth or quality of the information provided.

All participants were either CEOs or managed the operations/services within their organization. As we come on to explore, the weight of service sustainability does not impact all SVSSV service staff equally, with frontline practitioners often being “protected” from the full brunt of funding precarity by their managers. Almost half of our participants had worked in the specialist voluntary sector their entire career, progressing from roles such as advocate and helpline volunteer into managerial positions. For others, they had founded their service. The number of years spent working in one's organization ranged from one to 48 (with an average of 17.5 years), underscoring the experience and institutional memory captured within the sample.

Prior to each interview, CG explained—verbally and in writing—measures to ensure anonymity, confidentiality, data storage, and the voluntary nature of participation. Participants could retract their data up to 4 weeks after the interview and all provided signed consent. The study received institutional ethical approval and no ethical issues arose in respect to the disclosure of information or in relation to participants’ welfare. This likely reflects participants’ experience and skill at talking about the challenges of sustaining services and their conscious monitoring of the information they chose to share or otherwise (Appollis et al., 2015).

Interviews were digitally recorded and transcribed verbatim after they had been conducted. This allowed for the identification of lines of inquiry relevant to emergent theory which were pursued in subsequent interviews. Transcripts were anonymized and sense-checked against the original audio recordings by the interviewing researchers before being permanently deleted. Transcripts were read and coded initially by CG and 20% were verified with LI. During open coding (Strauss & Corbin, 1990) an inductive approach was taken where concepts and categories were allowed to emerge from the data. At this stage, it became apparent that service leaders’ ways of coping, resisting, and being affected by funding and commissioning practices were a central organizing concept within the data. It was also evident that participants used metaphor to describe these stresses, particularly metaphor which spoke to ideas of the “brink,” a boundary or edge. We harness this use of the edge, and the concept of Edgework (Lyng, 1990), recognizing its role in aiding communication and facilitating the “flow and exchange of experience” (Bauman, 1990, p. 231).

Following the development of a set of coded categories, transcripts were reread to ensure that codes accurately represented participants’ responses and to structure them in accordance with their relationships and subcategories. Selective coding allowed for a more detailed analysis of the core concept of edgework and its applicability to service leaders’ practice. Theory was enhanced and connections with codes were refined. The two overarching categories that emerged from this process were: “working at the edge” and “working with the edge,” which we set out below.

## Findings and Discussion

### Working at the Edge

Participants reported a range of positive relationships with individual commissioners and gave examples of progressive commissioner practice, commitment to the topic of sexual violence and those who saw and valued the expertise of the voluntary sector. However, different commissioners, often dependent on the commissioning body they worked for, were viewed as more or less engaged, knowledgeable, and willing to collaborate with SVSSV services. There was unanimous criticism of the broad commissioning agenda, with commissioning practices being described as “stressful,” causing “colossal burnout” (Participant 5. Hereafter P5) and having “implications … physiologically, psychologically, at all levels” (P2). As such, three subcategories were developed to capture the different but overlapping ways in which these pressures were pushing service leaders to a range of boundary points. These included, “the edge of surviving as a service versus no longer being financially sustainable,” “the edge of staying in the sector versus leaving” and “the edge of holding on psychologically versus going under emotionally.”

### The Edge of Surviving as a Service Versus No Longer Being Financially Sustainable

All participants experienced anxieties around the likely loss of funding and the potential for their service to close. Service leaders were acutely aware of the implications of this, that staff would lose jobs and survivors “a service that could be a lifeline” (P10)—implications that were said to be “terrifying.” While funding pressures will be felt by public and other voluntary sector managers, working with trauma and shame likely compounds these pressures (Horvath & Massey, 2018; Massey et al., 2019; Thornton & Novak, 2010). Indeed, violence against women and girls (VAWG) is not an attractive cause to support; it is still largely seen as a private problem and public perceptions around rape culture and victim blaming mean it does not garner the same sympathy as other social issues. This has been found to directly impact VAWG services’ ability to raise revenue, especially so for sexual violence services (see Barter et al., 2018).

Fears around sustainability were linked largely to the trajectory in England toward pooling commissioner budgets. That is, for commissioners in the same locality to combine funds in order to jointly tender for services and work toward shared outcomes (Home Office, 2022). While encouraging a more joined-up approach, it also creates a larger contract for organizations to bid for both in monetary value and expectations. Participants reported that in their localities this had increased competition. Not competition from other SVSSV services, although this was argued to have intensified, but from larger generic voluntary organizations and companies from the private sector: “…what I call the big boys will come in and take it (the service contract). Because it will be big enough then for it to be worthwhile. And also what they might think then is oh, we’ve got (area A). Perhaps now we’ll go for another area … and I think we will see the demise of specialist services…” (P13). For this participant, the architecture of commissioning posed a threat not just to individual service survival, but to the specialist voluntary sector as a whole. Fears that are well founded considering commissioning approaches for VAWG services have skewed the playing field to favor value for money over local knowledge, social value, and established community connections (Home Office, 2022). Indeed, participants reported that in those areas where this focus on value-dominated commissioning decisions, sexual violence specialism did not guarantee protection: “You could be the specialist sexual violence service but if somebody undercuts us, we could just lose it (the contract) just like that…” (P1). For one participant, the closure of their service loomed imminent due to recently having lost a key contract to a generic provider. As P1's comment implicates, the instability of operating on the edge of “losing it,” “just like that” posed a very real threat to participants and their sense of an ordered existence (Lyng, 1990).

Fears around service sustainability are also related to much sector funding being short-term and linked to innovations (APPG, 2018; WRC, 2018). This placed participants in a space of disorientation whereby “all the time you don’t know whether you can look forward or not” (P2). It frequently remained unclear, often until “the last minute” (P4), “whether services are going to continue or positions are going to continue or, you know, what's sustainable, what's not sustainable” (P9). The burden of continued application writing for pieces of short-term project money, as well as the protracted time required to respond to sexual violence service tender applications, was time-intensive and exhausting. It was also a “huge resource out of time with management that could be put into … the service” (P3). In smaller organizations with fewer staff, funding pressures were intensified and could only be balanced by deprioritizing other operational responsibilities: “I don’t have time to be inducting staff. I don’t have time to be training staff. All you’ve got the time for is to survive on a day-to-day basis” (P5). As others have argued, the pressures of responding to the commissioning and funding context can impact service quality, the ability to develop and action longer-term strategies, as well as political engagement. For example, campaigning and lobbying tend to be the activities that receive less attention, across U.S. and U.K.-based SVSSV services (Maier, 2011; Vacchelli et al., 2015). This is despite such activities serving important strategic purposes, as well as being an outlet for emotional offloading (Vera-Gray, 2020).

### The Edge of Staying in the Sector Versus Leaving

Two participants in our sample, each with decades of experience, were considering leaving their positions, while two others were debating taking early retirement. These decisions linked to the stresses discussed, however, they were also being driven by the perceived imposition of a commissioning agenda that jarred against the sector's, and participants’, longstanding values, ethos and ways of working: “I absolutely love working in the voluntary sector, it's where my heart is. But I don’t like what I see the specialist centers having to be in order to survive. And I think that's the question. What do we have to be? What are we willing to be in order to survive?” (P13).

Participants argued that commissioning had radically changed the way SVSSV services operated, moving them away from their synergy between survivor needs and the services delivered and toward a top-down approach driven by a commissioning agenda (APPG, 2018; WRC, 2018): “…There is a difference isn’t there when it's commissioned, because the council are then deciding these are the services we want to operate and this is what we’re prepared to pay for” (P7). When commissioners were developing local sexual violence service specifications, participants also argued that there was too little consultation with survivors or specialist service staff themselves: “The sector needs to be listened to about why we work in the way we do … it's about listening to service users and analyzing what's going on and seeing what works and why, and understanding the complexity of it…” (P7).

For participants, this was not about a nostalgic desire to return to a golden age of specialist voluntary sector work. Rather, the “Tesco-isation” (P13) of services, engendered through commissioning practices, meant that survivors were increasingly being fitted into standardized therapeutic options—typically 12 and at best 24 sessions of counseling. While participants recognized that this was often in response to commissioners wanting to decrease waiting list times and/or was due to cuts to their budgets, this streamlining of therapy moved services away from being truly “survivor-led organizations” (P8). Within this, there was also a perceived ushering in of a medical model-style approach to therapy. A style better suited to a finite number of counseling sessions and one where symptom stabilization and recovery as an end goal, as opposed to a process, was prioritized. Such an approach sits in tension with the original values of the sexual violence movement, through a shift in focus from “rape's social, cultural, and structural dimensions, to the individual and interpersonal” (Vera-Gray, 2020, p. 61). Consequently, certain participants felt that survivors were being directed into provisions that would not meet their needs:

You can’t work with those clients (those who have experienced long-term childhood abuse) in 3 to 6 months, you just can’t … what the commissioners will say—and I’ve had these arguments with them—is ‘Well you can only do 3 to 6 months. That's all the money that's available’, then they have to go back to their GPs and what they’re interested in is symptom reduction. So, can that person get up in the morning etc., etc.? And as long as that's OK, job done. I disagree. And what I think we’re doing is the voices of those survivors will not be heard. And I find that heartbreaking. So on the one hand we’re giving the message ‘Speak out’ and on the other hand we’re saying—excuse my French—‘Shut the fuck up’. (P13)

It was the top-down imposition of this changed practice and a broader undermining of “40 years of working in the field and listening to women” (P7) that challenged participants’ ethics and caused some to reconsider their futures in the sector. However, decisions to leave remained complex and contested. As Participant 13 notes, the voluntary sector is where “(her) heart is,” the same heart that breaks in response to the perceived inadequacy of provision. Those participants who spoke of leaving were typically on a bifurcation point, a boundary of “holding on” versus “letting go,” pulled between deep feelings of fatigue, care, commitment, frustration and an intensity to stay: “they have offered me, as I said, another 6 months and I keep thinking oh do I want another 6 months of this! And then I think, oh I can't bear to let it go” (P2).

### The Edge of Holding on Psychologically Versus Going Under Emotionally

As opposed to leaving the sector, or possibly a precursor to it, was the potential for “going under,” breaking down or experiencing other acute responses to the pressures of funding and commissioning arrangements. One participant talked openly about the mental health breakdown she had experienced, linked to wider life stresses but aligned with the demands of her role, particularly the stress of her service closing. A pressure mitigated only latterly:

I had a breakdown last year before Christmas. Strangely enough, we were due to close in September 2018, obviously I told everybody one by one, every client, I sat them down and explained and I got sort of through that phase, went to commissioners, went to CCGs begging … when the new money did come in it was for roles, which took me from managing a team and the clients and the assessments and everything else, to then managing two people on top of what I was already doing—then I had two deaths within a month … And I suddenly had all this going on and I just went under. (P6)

For this participant, her job pushes her to and over her mental limits, seemingly inflicting what Lyng (1990) would describe as an assault on her sense of control, causing her to lose balance. Thus, this was edgework that threatened mental well-being, that was harrowing and could result in the edge being breached. However, this did not mark the end for P6, who after several months returned to her role alongside a restructured set of responsibilities, whereby she would “let the service delivery go and stick to the paperwork.” A solution aimed at better preventing her, this second time, from losing her footing. The “thrill” here, or perhaps more appropriately, the compulsion to return to circumstances which may once again take her to the brink, does not lie in the sensory kick gained from navigating the edge—far from it—but rather, in response to the emotional ties and commitment she feels to her clients and service.

For those who had not so overtly fallen over the edge, the potential to do so was evident for some: “I used to enjoy it, the job, and now it's just sleepless nights and stress and waking up in the night thinking I just cannot, I can’t put one foot in front of the other” (P5). Here, the stress of the job leaves marks over time that sink into the participant, following her home and impacting her sleep. For others, for those whose “edge was further out” (Lois, 2001), or who constituted the “very niche selection of individuals who can survive in the environment” (P5), protracted feelings of overwhelm were less frequent. Stress, however, was still commonplace as was the potential for shorter-term, embodied responses that threatened composure:

The other one was the procurement phoned up and said ‘oh we've sent you the wrong link and the deadline's at five’, again I was away from the office, because you think you're allowed out after it's happened (submitting the funding application), and I was actually sick. Because the strain, the stress of it, I just thought I've got to get back before that deadline. (P11)

The ability to operate daily at such an emotional tenor would have been unsustainable. Rather, these psychological stresses were experienced more acutely at different points. Participants talked about “these waves of stress” (P13) and the “rhythms of the voluntary sector” (P11) and once they were understood it was less overwhelming. There were pinch points that placed pressure, particularly at the start of the calendar year when staff were waiting to hear back on funding applications and anxieties were intensified around whether services and positions would continue. Between April and June, these decisions were being made and the atmosphere was said to be lighter because budgets were known. Hence, for many, the intensity of the psychological edge—or participants’ closeness to it—was sharper at particular times of the year.

### Working With the Edge

Having set out the different edge points that SVSSV service leaders work at, we move on to examine the ways in which they navigate these boundaries. That is, the strategies they use to attempt to reinstate order over chaos. Three subcategories were developed: “circumventing the edge,” “emotion management,” and “remolding the edge.” We do not assume that these are the entirety of the approaches adopted but they represent those that appeared most often in the data.

### Circumventing the Edge

Edgework can serve as an escape from or resistance to the rules and routines of organizations, offering the means for autonomy and action. It is often an unspoken necessity conferred by organizations (or in this instance commissioners), who displace responsibility for risk and the management of inequalities onto individual employees and services (Lyng, 2009). Paradoxically then, edgework can free workers from, while binding them further into, contemporary working conditions. It is an identifiable aspect of the workplace, albeit one that is infrequently acknowledged (Worrall & Mawby, 2013).

Certain participants in our sample could be construed as taking risks in the execution of their roles. For example, a number told us that despite certain arrangements being in place contractually around the number of therapeutic counseling sessions a survivor would receive, to meet need, it was sometimes necessary to “not (be) totally strict” (P9) with those arrangements: “…we’re not just, ‘OK, you’ve had your sessions, that's it, you go’” (P8). As is well documented (Gunby et al., 2020; Hester & Lilley, 2018), the flexibility, “can-do” attitude and brute determination of the voluntary sector in ensuring service users get what they need, contextualizes its preparedness to find solutions: “So there was only 12 weeks, as you know, payment for 12 weeks work, so for the rest of the time they’re with you, you pick them up anyway, that's what you do” (P6). Often this meant using funding from other sources to support extra sessions and if clients had to be put back to the beginning of a waiting list, it sometimes involved filtering them into “the social group or come(ing) along to a coffee morning or have(ing) that additional support from the telephone helpline” (P8) to provide some provision until therapy resumed. Similarly, there were degrees of flexibility in working with clients who lived outside a commissioned contract area. Flexibility found to exist with other VAWG services (Coy et al., 2011): “We wouldn't necessarily turn someone—like say if someone came from (location) and they had a reason why the center in (location) for example it wasn’t the best fit for them … we’re not totally strict on that (P9).”

These acts of circumvention were not about sector leaders being reckless or flouting contractual obligations, but about deploying their skills when operating at the edge. By taking risks in the form of creative solutions, participants not only ensure that survivors get more of what they need, but service leaders retain a degree of agency and possible sense of authenticity by working closer to the original ethos and mission of the sector (Worrall & Mawby, 2013). In this sense, edgework can provide the means for maneuvre in a context where expectations around service delivery are imposed from above.

### Emotion Management

The management of emotions—self and others—was a key approach used to maintain control. In terms of managing one's own emotions, a range of strategies were adopted to help stay grounded, think clearly and not become overwhelmed. For example, prioritizing and using formal supervision to best effect. This in and of itself, however, was typically not enough and all participants reported the importance of making time for self-care, whether that be through the practice of “mindfulness and relaxation” (P4) or via “…some sense of physical release. Gardening, cooking, singing, shouting, running, yoga” (P10). For a minority, emotional stoicism (Lois, 2001), or a commitment to getting on with the job, to “do what you need to do” (P6) and just “keep going” (P5) was paramount. Underpinned, at times, by a certain degree of fatalism: “So yes, it does take its toll. It is what it is…” (P6). These practices typically allowed participants the space to remain mindful of and actively enforce their boundaries. This included leaving work at the office: “I do have a chunter occasionally at home (about work)! But yeah … when I leave here, I leave here” (P12). Although as discussed, this was not a boundary that could be maintained by all.

The edges produced by funding and commissioning practices were also navigated through managing the emotions of colleagues. This could be formal in approach and involve managers performing carefully calibrated emotional work to find answers to impossible questions: “I’ve had staff say to me, do you think that I should get a mortgage, or will I not be in a job in 2 years? And I almost can’t answer them” (P7). Similarly, in the execution of one's role, “emotional neutrality” (Ward et al., 2020), the suppression of one's true feelings and knowledge for the benefit of reassuring others, may be required: “We try to keep it away from the frontline workers (the precariousness of service survival) because they need to feel secure … so we kind of, we have to walk that line between letting everyone know because of HR responsibilities but continuing as normal. It's a challenge” (P11). Here, to work as a senior manager in an SVSSV service is to recognize one's responsibility to others by purposefully controlling emotions, so as to maintain the line between order and chaos. Emotions were also managed in less overt ways. For example, through the sense of camaraderie, care, and support that existed among colleagues: “Having that team around them is so crucial, like, in terms of sort of emotional, really supporting each other” (P9). As part of this, collective cynicism and humor had important roles to play. Both of these have the power to reinforce a sense of identity, team spirit, and self-esteem (Craun & Bourke, 2014). One participant told us about their organization keeping “a shit list” that included a record of broken web links to funding opportunities, as well as examples of jargonistic/inappropriate language used on related submission portals. The list, it could be argued, provides the means through which the absurdity of commissioning processes can be disassociated from, helping service leaders to remain, in certain moments, unaffected by them. To laugh with colleagues also serves to energize, regain a sense of power and release the pent-up stress long felt in relation to commissioning practices.

### Remolding the Edge

Several participants identified ways in which they attempted to make inroads into and shape the commissioning/funding status quo: “We’ve worked really hard, right from the beginning, to link into national organizations and sort of grabbed opportunities … I don’t really have time to do it but I will always prioritize it” (P1). Worrying about what the future holds compelled certain participants to make a plan of action, to prepare for edgework, by imagining the worst scenario and putting steps into place to try and influence otherwise (Lois, 2001; Lyng, 1990). This strategic work included involvement in stakeholder or commissioner groups that may be able to influence the local and national picture: “I also sit on a number of strategic groups that can influence how the area and different commissioning boards and bodies look at and approach and respond to sexual violence and abuse” (P10). Working with decision-makers on issues pertinent to survivors creates space for SVSSV leaders’ expertise to be heard and offers opportunities to inform and influence the decision-making process (Ishkanian, 2014). However, in services with fewer staff, this typically could not be facilitated, due, as described, to the focus on prioritizing income generation.

Being involved with umbrella organizations was also seen, by most, to be important strategic work that enabled managers to “feel like you’re not just alone” (P1) and to amplify the voices of individual organizations: “That's where organizations like The Survivors Trust and Rape Crisis England and Wales are important because sometimes they’re that go between” (P13). This was another means through which it was felt possible to influence the landscape, this time at a collective level. Opportunities to meet with colleagues nationally at events held by umbrella organizations were also described as invigorating, providing the means to raise concerns, jointly strategize and (emotionally) vent: “You are with … other center managers that have similar issues, similar struggles, so you do have somebody that you can go ‘what the hell, how come, why would they?’” (P12).

Diversifying funding streams so as not to be overly reliant on a select few funders—to spread the risk—was also something participants did: “So the need … to apply for diverse income, yeah, definitely increasing” (P10). Having a mix of commissioned contracts, grants from statutory agencies and project-based funding from charitable trusts and foundations was viewed as preferential. However, having “14 different sources … That's our funding pipeline” (P12) and in larger organizations, many more, came with multiple reporting expectations. Activities that again place pressure on management. The drive toward pooling local commissioner budgets was also reported to have shrunk the availability of different funding streams that could be applied to, reducing, for some, the capacity to adopt this approach.

In response to this context and trajectory, some participants were thinking more broadly (and radically) about sustainability in order to envisage an approach that could work for them. This included developing packages of specialist training to sell to other organizations in order to generate revenue. As part of this, moving the status of their service from a charity to a Charitable Incorporated Organization, which better supports income generation. For others, there were longer-term plans to acquire property, for example, to enable them to have less reliance on state funding and possibly, one day, be able to opt out of engaging with commissioning processes entirely. The potential for such, and for reenvisaging a better future for the sector, was the motivation that enabled control over current circumstances to be maintained:

Interviewer: How have you managed all of this (the pressures of funding and commissioning practices)?

Interviewee: By thinking about how we sustain ourselves in a different way … So using a different model and it's exciting, it's empowering, it's creative, it's a different way of thinking…. (P11)

## Conclusion

This article has offered an original way to think about and theorize SVSSV service leaders in the context of precarious funding and commissioning arrangements. It provides the first application of edgework to this group, contributing a broader understanding of the work leaders do, the impacts it can have and the vulnerability, as well as the agility, of this group of workers. In the process of financially sustaining their services—an aspect of the job often marginalized in academic accounts of the sector's stressors—service leaders become key players in controlling the boundary between order and chaos (Lyng, 1990). In ensuring their service financially survives, that staff retain their jobs and survivors receive a crucial source of support, sector leaders are pushed to their physical and emotional limits. The pressure from which, in combination with the compromises commissioning and funding practices necessitate, push leaders to—and sometimes over—the edge of emotional well-being and job satisfaction.

Although we argue that working with stigma and shame creates unique sustainability challenges for sexual violence services, we suggest that aspects of our findings may be translatable to senior leaders working in other areas of the voluntary sector—in England and elsewhere—and would encourage future research to explore this possibility. This seems particularly relevant to those leaders working in specialist services for Black and Minoritized women. Considering their especially precarious financial position (Imkaan, 2016; WRC, 2020), and the intersecting forms of oppression they work with, this is imperative. We also recommend applying the lens of edgework to different groups of voluntary sector workers, particularly frontline practitioners. Although our data suggests frontline staff in SVSSV services are protected from the full force of service precarity, this claim is not borne out of evidence. Further research, within and outside SVSSV services, would help to substantiate the claim (or otherwise). Such research would also elucidate the different forms of edgework occurring within voluntary services, the agendas that motivate them and the degree to which risk-taking has become a necessity to enable commissioned contracts to be delivered. Such insights will expand and refine the concept of edgework and understandings, more broadly, around its application to workers within the voluntary sector.

However, for SVSSV leaders, working at the edge, as well as returning to it once crossed, is not due to the pursuit of a sensory “high,” but borne out of a commitment to one's service, its staff and the individuals who use it. We have emphasized the routine nature of edgework for this group, the necessity to enact it to offset detrimental aspects of commissioning, challenged its traditional associations with pleasure and underscored its relationship to emotional pain (Ward et al., 2020). In so doing, we have expanded the types of edges that count as edgework.

Our analysis emphasizes what work from the sector has long been saying. That too frequently, funding and commissioning frameworks do not work in ways that allow for the healthy sustainability of SVSSV services and the workers within them. In order to work better, the dynamics of commissioning must be changed to allow those within the sector—who acutely understand the needs of their communities—to use that expertise to help define funding priorities, the services to be delivered and the structures for delivering them. The feminist solution for improving the current status quo—a solution that we advocate—calls for commissioners and funders to make it easier for SVSSV services, including minoritized women services, to work in partnership to build capacity and strengthen structures for referral and multiagency working. Such approaches would serve to promote unity over competition, collaboration in the envisaging of new approaches to service sustainability and exploration of formal partnership/consortia working to expand the size, scope, reach, intersectional approach, and voice of specialist provision (Barter et al., 2018; Home Office, 2022; Miller & Jones, 2019; Thiara & Roy, 2020; Vacchelli et al., 2015; WRC, n.d). This fits with a more “ecosystem” based approach to the resourcing of services. The aims of which are for individuals and organizations to interact in more connected and complementary ways, after all players, but particularly funders/commissioners, have first asked “what role can I play in complementing the overall priorities and resourcing of this movement?” (Arutyunova, 2018; Miller & Jones, 2019). Indeed, a crucial step in improving funding and commissioning dynamics is to redress the current imbalance of power, a particularly pertinent shift in the sexual violence sector, given the role disempowerment plays in the perpetration and experience of sexual abuse.

As well as illuminating service leaders’ struggles with state approaches to the resourcing of SVSSV services, our data have highlighted the strategies, infrequently passive, that are used to find the means to ease, cope with, or remold those pressures. Echoing Miller and Jones (2019, p. 10), while funding is integral to the sustainability of specialist voluntary services, power is also evidenced through “daily acts of resistance, care, survival, and building new feminist realities” which can help, in their own right, to sustain organizations and individuals. Such acts were abundant in our data and point toward a range of practices that can support a holistic approach to sustainable services. For example, the importance of routine formal supervision, access to supportive colleagues and professional networks, or umbrella organizations, which can act as an outlet for discussing work, letting off emotional steam, building camaraderie, sharing experiences and good practice. Further, role-specific training, routine monitoring of staff well-being and supported schemes to aid coping mechanisms all appear warranted (Horvath et al., 2021), especially at those points of the year when funding pressures are intensified and individuals find themselves closer to the edge. These elements—more often controlled within services—will help, as our data indicates, to mitigate some of the impact of external funding and commissioning pressures.
